# Individual Copy Number of Ribosomal Genes as a Factor of Mental Retardation and Autism Risk and Severity

**DOI:** 10.3390/cells8101151

**Published:** 2019-09-26

**Authors:** Lev Porokhovnik

**Affiliations:** Research Centre for Medical Genetics, 1 Moskvorechie str., Moscow 115478, Russia; med-gen@mail.ru

**Keywords:** autism, ribosome, rDNA, ribosomal gene, TSC, copy number

## Abstract

Autism is a complex multifactorial developmental disorder characterized by deficits in communication and restricted interests, often followed by mental retardation. Autism spectrum disorders (ASD) are caused by defects in miscellaneous molecular mechanisms, many of which remain unclear. But a considerable part of the known pathways converges on protein synthesis or degradation processes at different stages in the dendrites, laying the foundation for a concept of disturbed “translational homeostasis” or “proteostasis” in autism. The protein synthesis is conducted on ribosomes, cellular organelles consisting from a complex of riboproteins and a ribosomal RNA (rRNA) framework. The rRNA is encoded by ribosomal genes (RG) existing in multiple copies in the genome. The more copies of RG that are contained in the genome, the higher is the peak (maximum possible) ribosome abundance in the cell. A hypothesis is proposed that the RG copy number, through determining the quantity of ribosomes available in the dendrites, modulates the level of local dendritic translation and thus is a factor of risk and severity of a series of neuropsychiatric disorders caused by aberrant dendritic translation. A carrier of very low copy number of ribosomal genes is expected to have a milder form of ASD than a subject with the same epigenetic and genetic background, but a higher ribosomal gene dosage. Various ways of evaluation and testing the hypothesis on clinical material and animal models are suggested.

## 1. Introduction

**Figure 1:**
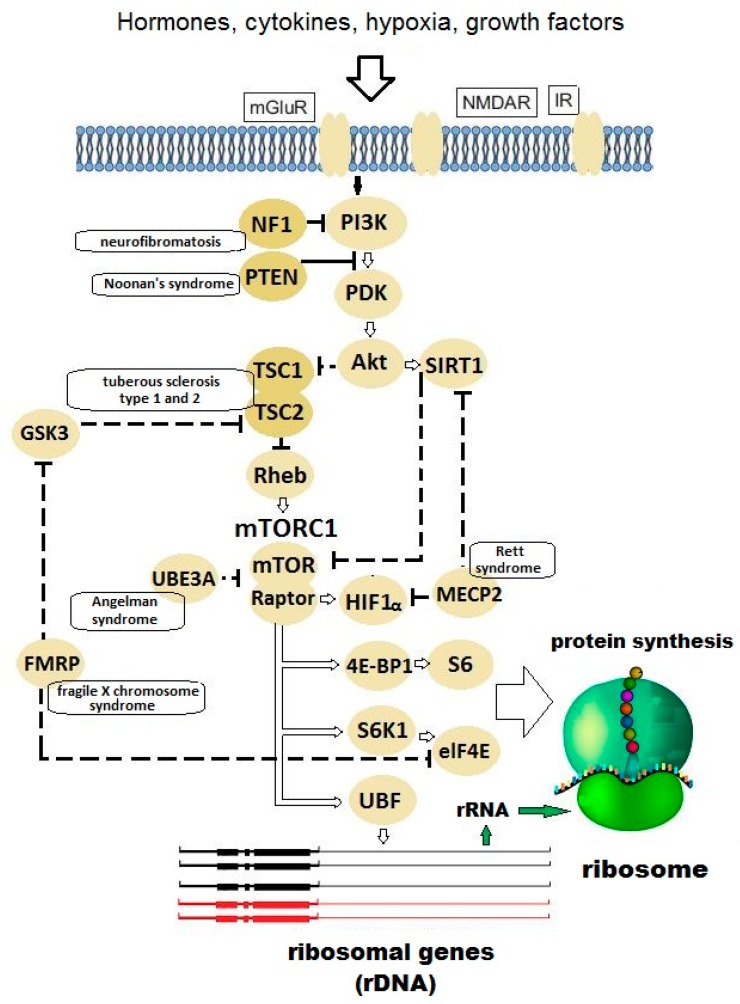
The molecular signaling pathways that control the pace of protein biosynthesis in ribosomes. An arrow stands for activation or synthesis of a product, a dashed line means suppression. Close to the key regulatory proteins, monogenic syndromes are indicated, which result from mutations in the gene for the relevant protein and cause mental retardation and autism in most (but not all) cases. Abbreviations: mGLuR—metabotropic glutamate receptor; NMDAR—N-methyl-D-aspartate receptor; IR—insulin receptor; NF1—neurofibromin; PI3K—phosphoinositide-3 kinase; PTEN—phosphatase and tensin homolog deleted on chromosome 10; PDK—phosphoinositide-dependent kinase; TSC1/2—tuberous sclerosis complex 1/2; Akt—protein kinase B; SIRT1—sirtuin 1; Rheb—rat sarcoma homolog enriched in brain; GSK3—glycogen synthase kinase 3; mTORC1—mammalian target of rapamycin complex 1; mTOR—mammalian target of rapamycin; Raptor—regulatory-associated protein of mTOR; UBE3A—ubiquitin protein ligase E3A; HIF1α—hypoxia-inducible factor 1-alpha; MECP2—methyl-CpG binding protein 2; FMRP—fragile X mental retardation protein; eIF4E—eukaryotic initiation factor 4E; 4E-BP1—4E-binding protein 1; S6—ribosomal protein S6; S6K1—p70 ribosomal S6 kinase; UBF—upstream binding factor.

Autism is a complex multifactorial disease associated with >1000 genes. For example, the SFARI (Simons Foundation Autism Research Initiative) database currently lists 1036 genes implicated in autism [1]. Approximately 31% of patients with autism and ASD (autism spectrum disorders) present mental retardation, also termed intellectual disability (reduced communication and learning capacities, IQ < 70) [2]. Autism and mental retardation are caused by various molecular mechanisms, many of which remain unclear. But a considerable part of known pathways converges on protein synthesis or degradation processes (Figure 1) at different stages laying the foundation for a concept of disturbed “translational homeostasis” or “proteostasis” in ASD [3,4,5].

Essential for studying and understanding the ASD pathogenesis are several monogenic syndromes with high autism rates (see Table 1) [6]. Among them, most frequent is fragile X syndrome caused by the lack of family mental retardation protein (FMRP) encoded by FMR1 gene. The FMRP functions as a translation repressor [7,8,9] for a certain class of mRNA.

Animal models have been developed for most common monogenic forms of autism in laboratory rodents, zebrafish (*Danio rerio*) and *Drosophila*. Animal model studies help forward a fundamental understanding of the pathogenic mechanism and search for promising agents of pharmacologic correction [4,10,11,12].

The existing outline of pathogenesis posits that exaggerated protein synthesis affects synaptic plasticity and cognition through the disturbed mechanism of local dendritic translation, because the transition from labile short-term memory to stable long-term one requires a transient burst of synthesis of specific proteins in dendrites [6,13].

It remains unclear, however, why mental retardation and autism are not intrinsic in every case of the monogenic syndromes (see Table 1, the fourth column). So, tuberous sclerosis is known to have so-called “formes frustes” (from French “crude forms”, or “unfinished forms”) with safe or slightly affected intellect. These forms are often misdiagnosed [14,15] or remain non-diagnosed until an affected child is born. The only known cause of formes frustes is genetic mosaicism [16,17,18], but it covers just a part of the phenomenon cases. Finding the answer as to why the intellect is rescued in some cases of syndromic autism could shed the light on the autism pathogenesis and possible ways to prevent its progression.

Defects of memory, learning, cognition and communication can be caused by mutations in genes that control different stages of protein synthesis: Initiation (*eIF2d*, *eIF4e*) [19,20], poly (A), the mechanistic target of rapamycin complex 1 (*mTORC1*), elongation (*eEF2*), folding (reviewed in: [21]). The final loop of the translation chain is the ribosome, and there are known autism-associated “ribosomopathies”, i.e., defects in riboproteins (e.g., disrupted Rpl10) [22,23,24,25], but no attention is paid to such factors as the abundance (quantity) of available normal ribosomes limited by the availability of ribosomal RNA (rRNA), the major component of the ribosome, encoded by ribosomal genes.

Due to high demand for rRNA (~60–80% of total cellular RNA), the ribosomal genes are represented by tandem repeats in the genome of every eukaryotic organism. Human ribosomal RNA genes encoding a pre-transcript of the three major ribosomal RNA (18S, 5.8S and 28S rRNA) are clustered in five pairs of human chromosomes (13, 14, 15, 21, 22). According to our studies, the total copy number of rRNA genes varies from 250 to 670 per diploid genome in different individuals with a mean of approximately 420 copies, but only a fraction of them is transcriptionally active [26]. There is a recent study with a different estimate [27], but the studies concur to report significant inter-individual variations in rDNA copy numbers. The functional consequences of human rRNA gene dosage are not widely known and often assumed to be negligible. However, there is a body of facts that individual rRNA gene dosage has an effect on normal growth and ageing, stress resistance of healthy subjects and survivability of patients with chromosomal abnormalities, as well as on the risk and severity of some multifactorial diseases with proven genetic predisposition (reviewed in: [26]).

Obviously, the genome dosage of human ribosomal genes that determines the overall ribosomal number [28,29] and hence limits protein synthesis and growth [30,31], should be expected to have an influence on the pace of protein synthesis in dendrites during key moments of synapse formation thus modulating the progression of mental diseases associated with exaggerated translational homeostasis.

## 2. Hypothesis

**Figure 2:**
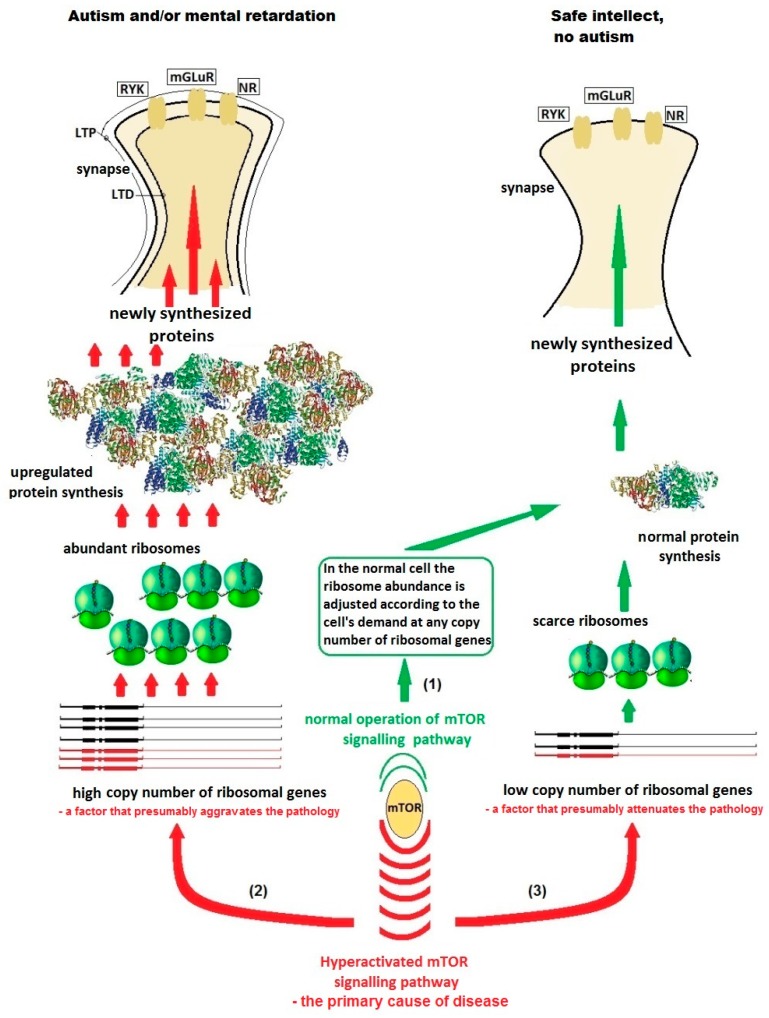
A hypothetical scheme of the influence of ribosomal gene (RG) dosage in the genome of a non-carrier (green text and arrows) and a carrier (red text and arrows) of a monogenic autism mutation on the disease severity. The idea is that low RG copy numbers that lead to ribosome underabundance would attenuate the signs of monogenic autism caused by excessive protein production in the dendrites. (1) Without mutation, the copy number of ribosomal genes makes no difference. The cell produces such quantity of ribosomes and proteins as required for its functioning at the moment, via switching on and off the ribosomal DNA (rDNA) transcription according to demand. (2) A mutation resulting in hyper-activation of mTOR signaling pathway, provided that the cell has a sufficient quantity of ribosomal genes in the genome, leads to elevated rDNA transcription and hyper-activation of local (dendritic) translation resulting in overproduction of some proteins. The protein overproduction results in aberrant synapse morphology and defect LTD/LTP processes in the synaptic networks. Autism and (or) mental retardation develops. (3) I hypothesize here that formes frustes of the monogenic syndromes with safe intellect are only possible in case of the presence of a small number of copies of ribosomal genes in the genome. In that case, the ribosomal genes, despite hyperactivation, are unable to produce so much rRNA and, consequently, ribosomes, so that the local protein synthesis in dendrites would be measurably disturbed. Abbreviations: LTD/LTP—long-term depression/potentiation; RYK—receptor-like tyrosine kinase; mGLuR—metabotropic glutamate receptor; NR—N-methyl-d-aspartate receptor.

I postulate here that the copy number of ribosomal genes (genes encoding rRNA) through determining the available ribosome abundance in the dendrites modulates the level of local dendritic translation and thus is a factor of risk and severity of a series of neuropsychiatric disorders caused by aberrant dendritic translation (Figure 2).

Within this scheme, autism and intellectual disability will develop in subjects with dysregulated translation on abundant ribosomes owing to moderate or high copy numbers of ribosomal genes (genes for rRNA), whereas those subjects, whose genomes contain very few genes for rRNA (but still sufficiently for normal life, of course), will have normal intellect despite the same genetic defect.

## 3. Evaluation of Hypothesis

First of all, the following question should be addressed: Why has this idea not been proposed ever before?

The scientific community is poorly aware of the variability of ribosomal gene copy number in man and the effects of this trait. Ribosomal RNA remains in the unassembled part of human genome after the completion of Human Genome project. There are only rare publications in this field with calls for studying the manifestation of the individual ribosomal DNA (rDNA) copy number [27]. That’s why the researchers’ attention is focused on upstream signaling, not on the quantitative factor of ribosome abundance.

Empirical data that could prove the hypothesis are indirect and segmentary. They are listed below.

(a) Autism and ASD are known to have some similarity with schizophrenia [32], a mental disorder characterized by abnormal behavior, strange speech and a decreased ability to understand reality, unclear or confused thinking, reduced social engagement and emotional expression and lack of motivation, with a global prevalence of the same magnitude as autism [33,34]. Schizophrenia patients have an elevated rDNA abundance in their genomes [35]. This fact can point at an exaggerated protein synthesis and distorted translational homeostasis in the patients.

(b) There are some anecdotal reports from practical neurologists (personal communications to the author) that the formes frustes of tuberous sclerosis with safe intellect are more affected somatically. This observation can be well explained by the decreased rDNA content in such cases, because subjects with low copy numbers of ribosomal genes are generally more amenable to stresses [26], and the same is true of *Drosophila* “bobbed” phenotype (see below in the “Predictions” section, subparagraph (ii) of paragraph b).

(c) There is a body of cases of ameliorating symptoms and behavior in some children with autistic disorders during and/or soon after a heat shock, such as fever or external heat (e.g., sauna) [36]. Special therapeutic devices called “heat tubes” or infrared saunas have been developed and, though unapproved officially for autism correction and often criticized, are offered for the treatment of responders as a device for “toxin clearance” [37,38]. Scientifically, the effect of heat shock was hypothetically attributed to different factors, including altered expression of the causative genes [39], or transiently restoring the modulatory functions of the locus coeruleus-noradrenergic (LC-NA) system [40], or the effect of stress-induced chaperons termed heat-shock proteins on proteostasis [41,42]. An alternative explanation can be proposed on the base of the fact that the heat shock represses rRNA synthesis by inactivation of TIF-IA and lncRNA-dependent changes in nucleosome positioning [43]. Obviously, attenuation of RNA Pol I is a condition that simulates a genome with low number of active ribosomal genes: In both cases, the rate of rRNA production is low and this improves the cases with pathologically up-regulated translation.

## 4. Predictions from the Hypothesis and Direct Testing

Determining predictions and testing their consequences is the best way to prove a hypothesis.

My hypothesis predicts that the carriers of the “monogenic autism” mutations (neurofibromatosis, tuberous sclerosis, Cowden syndrome, and some other) in mild form with safe intellect have lower numbers of ribosomal genes in their genomes compared to intellectually disabled patients with the same syndromes, because scarce ribosomes are expected to attenuate the exaggerated dendritic translation in cases. The same can be referred to in animal models. This prediction naturally determines two directions of testing the hypothesis.

### 4.1. Testing on Patients

This way implies direct determination of the copy number of total and active (non-methylated) ribosomal genes in formes frustes and intellectually disabled cases of monogenic autism syndromes. The logically predicted result from the hypothesis is that subjects with safe intellect will show less copies of total and active ribosomal genes in their genomes. Alternatively, severe cases are expected to have higher rDNA abundance, because high ribosome content facilitates translations and thus aggravates the disease. Cases of mosaicism should be excluded from these tests. 

### 4.2. Testing on Animal Models

(*i*) Rodents: In a murine autism model, pharmacologic attenuation of RNA Pol I (like heat-shock treatment described above) would yield a decrease in ribosome content, thus simulating a very low dosage of active ribosomal genes. If the hypothesis is true, this will result in an improvement of autism-like symptoms and an increase in memory and learning abilities. Many agents for RNA I attenuation are available now. A series of novel rRNA synthesis inhibitors, such as quarfloxin [44], were developed recently.

(*ii*) *Drosophila*: Fruit fly is an ideal model for studying the effects of ribosomal gene dosage. Unlike human rDNA (ribosomal genes), which is distributed over five pairs of acrocentrics, *Drosophila* has only one pair of rDNA clusters on sexual chromosomes [45]. The rDNA cluster size is normally constant within the population and through generations because meiotic recombination in the rDNA arrays is rare due to their heterochromatic locations. However, there are genetically altered flies with partially deleted rDNA clusters, but are still sufficient for life. This rDNA deficiency determines the hypomorphic “bobbed” (*bb*) phenotype, described by Bridges [46] as a recessive trait characterized by slow development, production of short and thin thoracic bristles, thinning, and, sometimes, etching of the abdominal cuticular tergites and by deposition of dechorionated eggs. All of these morphological features reflect the reduced protein synthetic ability caused by lack of rRNA [47].

Despite the obvious anatomical difference, biological processes are highly conserved between *Drosophila* and humans at the molecular, cellular, and synaptic levels. About 75% of human disease genes have identifiable homologs in *Drosophila*, 44% of which are sufficiently conserved for functional study [48,49,50,51,52]. The effects of five of the most promising and probable ASD candidate genes: Fmr1 (fragile X mental retardation 1, FraX), Ube3a (ubiquitin-protein ligase E3A), Nrx-1 (neurexin-1), Nlg4 (neuroligin 4), and Tsc1 (tuberous sclerosis complex 1) are well investigated on *Drosophila* [53]. The *Drosophila* studies that characterize the molecular and behavioral abnormalities in fly ASD candidate gene mutants, including locomotor activity, memory and learning, and courtesy patterns are well reviewed in [54]. The most affected behavioral pattern is courtship in male flies [55]. Proceeding from the above-mentioned hypothesis, one can assume that double mutants with *bobbed* phenotype and simultaneously *fmr1* or *tsc1* autism gene ortholog mutations would have less affected memory/courtship pattern than the autism gene mutants without *bobbed* deletion of the rDNA cluster.

An original experiment design developed by Chang et al. [10] could be also useful for experimental testing. Wild type flies grew well on a medium containing glutamate, whereas *Fmr1* mutants died because of neuronal excitotoxicity. It can be predicted that *Fmr1* mutant flies with an additional *bobbed* deletion (double mutants) would have a higher threshold of glutamate excitotoxicity and less mortality on the glutamate medium.

## 5. Discussion

Monogenic autism cases constitute a mere part of ASD cases. However, I consider it possible that the hypothetic scheme represented above can relate to most, if not all, cases with idiopathic autism/ASD. So, the syndromic forms of autism are believed to account for up to 25% of the total autism spectrum cases [56]; however, microarray analysis of peripheral blood from patients with idiopathic autism has shown an abnormal activity of the signaling pathways associated with translation initiation [57]; and a direct measurement of Akt/mTOR pathway in vitro in blood cells isolated from children with idiopathic autism demonstrated elevated Akt/mTOR pathway activity as compared to healthy controls, suggesting an essential role of the mTOR pathway in the pathogenesis of the entire autism spectrum [58]. There is tentative evidence that hyperactivation of the Akt/mTOR pathway underpins approximately 80% of idiopathic ASD cases [59]. Dysregulation of the mTOR pathway has been identified in multiple models, suggesting a potential common mechanism in the development of ASD [60]. Perhaps, the exaggerated translation in idiopathic autism is realized through epigenetic mechanisms. Anyway, in most cases, a carrier of a very low copy number of ribosomal genes is expected to have a milder form of ASD or no signs at all, than a subject with the same epigenetic and/or genetic background, but a higher ribosomal gene dosage.

If the hypothesis is proved, two benefits can be obtained. The first one is a good prognostic possibility for family risk and de novo cases. Second, novel drugs targeting the rDNA transcription and the ribosome itself could be developed and applied as alternative therapy in psychiatry and neurology. There are already some reports of mood stabilizing effects of macrolids that inhibit the mitochondrial ribosomes, and rapamycin, which is an upstream inhibitor of cytoplasmic ribosomes via mTOR pathway blockage [61]. A possibility to tune precisely the activity of cytoplasmic ribosomes might become a promising step forward in the psychiatry.

I note finally, that the hypothesis is not relevant to any case of ribosomopathy, that is disrupted riboproteins [22,23,24,25], nor to defects in specific rRNA modifications required for rRNA biogenesis and assembly, playing a role in neocortical development and involved in some diseases (e.g., as reviewed in [62]). The hypothesis stated above comprises only a quantitative mechanism of modulating a proteostasis shift in cases with upregulated dendritic translation, which implies normal ribosomes.

## Figures and Tables

**Table 1:** Monogenic disorders characterized by a high frequency of autism (syndromic forms of autism).

Gene	Gene Function	Disease	Frequency of Autism in the Disease	Frequency of the Disease in All Autism Cases
1	2	3	4	5
*FMR1*	Translation repressor	Fragile X syndrome	15–30%	2–5%
*TSC1*/*2*	mTOR inhibitor	Tuberous sclerosis 1/2	25–60%	1–4%
*PTEN*	PI3K/mTOR signaling pathway inhibitor	Cowden syndrome	No data	1%
*NF1*	Ras-GTPase activating protein	Neurofibromatosis 1	4%	0–4%
*MECP2*	Global translation repressor	Rett syndrome	100%	2%
*UBE3A*	E3 ubiquitin ligase	Angelman syndrome	40%	1%
*NLGN3*/*4*	Synaptic adhesion	Familial ASD	No data	<1%
*NRXN1*	Synaptic adhesion	Familial ASD	No data	<1%

## References

[1] Simons Foundation Autism Research Initiative Base. https://www.sfari.org/resource/sfari-base/.

[2] Baio J., Wiggins L., Christensen D.L., Maenner M.J., Daniels J., Warren Z., Kurzius-Spencer M., Zahorodny W., Robinson-Rosenberg C., White T. (2018). Prevalence of Autism Spectrum Disorder Among Children Aged 8 Years - Autism and Developmental Disabilities Monitoring Network, 11 Sites, United States, 2014. MMWR Surveill Summ..

[3] Richter J.D., Bassell G.J., Klann E. (2015). Dysregulation and restoration of translational homeostasis in fragile X syndrome. Nat. Rev. Neurosci..

[4] Bhattacharya A., Mamcarz M., Mullins C., Choudhury A., Boyle R.G., Smith D.G., Walker D.W., Klann E. (2016). Targeting Translation Control with p70 S6 Kinase 1 Inhibitors to Reverse Phenotypes in Fragile X Syndrome Mice. Neuropsychopharmacology.

[5] Hinnebusch A.G. (2012). Translational homeostasis *via* eIF4E and 4E-BP1. Mol. Cell.

[6] Kelleher R.J., Bear M.F. (2008). The autistic neuron: Troubled translation?. Cell.

[7] Laggerbauer B., Ostareck D., Keidel E.M., Ostareck-Lederer A., Fischer U. (2001). Evidence that fragile X mental retardation protein is a negative regulator of translation. Hum. Mol. Genet..

[8] Chen E., Joseph S. (2015). Fragile X mental retardation protein: A paradigm for translational control by RNA-binding proteins. Biochimie.

[9] Ciaccio C., Fontana L., Milani D., Tabano S., Miozzo M., Esposito S. (2017). Fragile X syndrome: A review of clinical and molecular diagnoses. Ital. J. Pediatr..

[10] Chang S., Bray S.M., Li Z., Zarnescu D.C., He C., Jin P., Warren S.T. (2008). Identification of small molecules rescuing fragile X syndrome phenotypes in *Drosophila*. Nat. Chem. Biol..

[11] Bey A.L., Jiang Y.H. (2014). Overview of mouse models of autism spectrum disorders. Curr. Protoc. Pharmacol..

[12] Chen J., Lei L., Tian L., Hou F., Roper C., Ge X., Zhao Y., Chen Y., Dong Q., Tanguay R.L. (2018). Developmental and behavioral alterations in zebrafish embryonically exposed to valproic acid (VPA): An aquatic model for autism. Neurotoxicol. Teratol..

[13] Costa-Mattioli M., Sossin W.S., Klann E., Sonenberg N. (2009). Translational control of long-lasting synaptic plasticity and memory. Neuron.

[14] Gumbinger C., Rohsbach C.B., Schulze-Bonhage A., Korinthenberg R., Zentner J., Häffner M., Fauser S. (2009). Focal cortical dysplasia: A genotype-phenotype analysis of polymorphisms and mutations in the TSC genes. Epilepsia.

[15] Kerr L.A., Blute M.L., Ryu J.H., Swensen S.J., Malek R.S. (1993). Renal angiomyolipoma in association with pulmonary lymphangioleiomyomatosis: Forme fruste of tuberous sclerosis?. Urology.

[16] Kwiatkowska J., Wigowska-Sowinska J., Napierala D., Slomski R., Kwiatkowski D.J. (1999). Mosaicism in tuberous sclerosis as a potential cause of the failure of molecular diagnosis. N. Engl. J. Med..

[17] Curatolo P., Moavero R., Roberto D., Graziola F. (2015). Genotype/Phenotype Correlations in Tuberous Sclerosis Complex. Semin. Pediatr. Neurol..

[18] Byers H.M., Jensen D.M., Glass I.A., Bennett J.T. (2018). Minimal mosaicism, maximal phenotype: Discordance between clinical and molecular findings in two patients with tuberous sclerosis. Am. J. Med. Genet. C Semin. Med. Genet..

[19] Gkogkas C.G., Khoutorsky A., Ran I., Rampakakis E., Nevarko T., Weatherill D.B., Vasuta C., Yee S., Truitt M., Dallaire P. (2012). Autism-related deficits *via* dysregulated eIF4E-dependent translational control. Nature.

[20] Santini E., Huynh T.N., MacAskill A.F., Carter A.G., Pierre P., Ruggero D., Kaphzan H., Klann E. (2012). Exaggerated translation causes synaptic and behavioural aberrations associated with autism. Nature.

[21] Buffington S.A., Huang W., Costa-Mattioli M. (2014). Translational control in synaptic plasticity and cognitive dysfunction. Annu. Rev. Neurosci..

[22] Klauck S.M., Felder B., Kolb-Kokocinski A., Schuster C., Chiocchetti A., Schupp I., Wellenreuther R., Schmötzer G., Poustka F., Breitenbach-Koller L. (2006). Mutations in the ribosomal protein gene RPL10 suggest a novel modulating disease mechanism for autism. Mol. Psychiatry.

[23] Chiocchetti A., Pakalapati G., Duketis E., Wiemann S., Poustka A., Poustka F., Klauck S.M. (2011). Mutation and expression analyses of the ribosomal protein gene RPL10 in an extended German sample of patients with autism spectrum disorder. Am. J. Med. Genet. A.

[24] Alsop R.J., Soomro A., Zhang Y., Pieterse M., Fatona A., Dej K., Rheinstädter M.C. (2016). Structural Abnormalities in the Hair of a Patient with a Novel Ribosomopathy. PLoS ONE.

[25] Paolini N.A., Attwood M., Sondalle S.B., Vieira C.M.D.S., van Adrichem A.M., di Summa F.M., O’Donohue M.F., Gleizes P.E., Rachuri S., Briggs J.W. (2017). A Ribosomopathy Reveals Decoding Defective Ribosomes Driving Human Dysmorphism. Am. J. Hum. Genet..

[26] Porokhovnik L.N., Lyapunova N.A. (2018). Dosage effects of human ribosomal genes (rDNA) in health and disease. Chromosome Res..

[27] Gibbons J.G., Branco A.T., Yu S., Lemos B. (2014). Ribosomal DNA copy number is coupled with gene expression variation and mitochondrial abundance in humans. Nat. Commun..

[28] Larson D.E., Zahradka P., Sells B.H. (1991). Control points in eucaryotic ribosome biogenesis. Biochem. Cell Biol..

[29] Likhoshvai V.A., Kogai V.V., Fadeev S.I., Khlebodarova T.M. (2016). Chaos and Hyperchaos in a Model of Ribosome Autocatalytic Synthesis. Sci. Rep..

[30] Von der Haar T. (2008). A quantitative estimation of the global translational activity in logarithmically growing yeast cells. BMC Syst. Biol..

[31] Perry R.P. (2007). Balanced production of ribosomal proteins. Gene.

[32] Gao R., Penzes P. (2015). Common mechanisms of excitatory and inhibitory imbalance in schizophrenia and autism spectrum disorders. Curr. Mol. Med..

[33] Bhugra D. (2005). The global prevalence of schizophrenia. Plos Med..

[34] Lai M.C., Lombardo M.V., Baron-Cohen S. (2014). Autism. Lancet.

[35] Chestkov I.V., Jestkova E.M., Ershova E.S., Golimbet V.E., Lezheiko T.V., Kolesina N.Y., Porokhovnik L.N., Lyapunova N.A., Izhevskaya V.L., Kutsev S.I. (2018). Abundance of ribosomal RNA gene copies in the genomes of schizophrenia patients. Schizophr. Res..

[36] Curran L.K., Newschaffer C.J., Lee L.C., Crawford S.O., Johnston M.V., Zimmerman A.W. (2007). Behaviors associated with fever in children with autism spectrum disorders. Pediatrics.

[37] Beyond Health® vitamin and supplement supplier’s corporate website. http://www.beyondhealthnews.com/wpnews/index.php/2013/03/far-infrared-saunas-help-reverse-autism/.

[38] (2012). Forbes Magazine, Issue November 5. https://www.forbes.com/sites/emilywillingham/2012/11/05/we-can-now-add-forced-sweating-to-the-faux-autism-treatment-list/#362969e0369f.

[39] Lin M., Zhao D., Hrabovsky A., Pedrosa E., Zheng D., Lachman H.M. (2014). Heat shock alters the expression of schizophrenia and autism candidate genes in an induced pluripotent stem cell model of the human telencephalon. Plos ONE.

[40] Mehler M.F., Purpura D.P. (2008). Autism, fever, epigenetics and the locus coeruleus. Brain Res. Rev..

[41] Di Salvo E., Casciaro M., Quartuccio S., Genovese L., Gangemi S. (2018). Do Alarmins Have a Potential Role in Autism Spectrum Disorders Pathogenesis and Progression?. Biomolecules.

[42] Singh K., Connors S.L., Macklin E.A., Smith K.D., Fahey J.W., Talalay P., Zimmerman A.W. (2014). Sulforaphane treatment of autism spectrum disorder (ASD). Proc. Natl. Acad. Sci. USA.

[43] Zhao Z., Dammert M.A., Hoppe S., Bierhoff H., Grummt I. (2016). Heat shock represses rRNA synthesis by inactivation of TIF-IA and lncRNA-dependent changes in nucleosome positioning. Nucleic Acids Res..

[44] Hald Ø.H., Olsen L., Gallo-Oller G., Elfman L.H.M., Løkke C., Kogner P., Sveinbjörnsson B., Flægstad T., Johnsen J.I., Einvik C. (2018). Inhibitors of ribosome biogenesis repress the growth of MYCN-amplified neuroblastoma. Oncogene.

[45] Ritossa F.M., Ashburner M., Novitski E. (1976). The bobbed locus. The Genetics and Biology of Drosophila.

[46] Bridges C.B. (1916). Non-disjunction as a proof of the chromosome theory of heredity. Genetics.

[47] Ritossa F., Atwood K.C., Spiegelman S. (1966). A molecular explanation of the bobbed mutants of *Drosophila* as partial deficiencies of “ribosomal” DNA. Genetics.

[48] Reiter L.T., Potocki L., Chien S., Gribskov M., Bier E. (2001). A systematic analysis of human disease-associated gene sequences in *Drosophila melanogaster*. Genome Res..

[49] Adams M.D., Celniker S.E., Holt R.A., Evans C.A., Gocayne J.D., Amanatides P.G., Scherer S.E., Li P.W., Hoskins R.A., Galle R.F. (2000). The genome sequence of *Drosopila melanogaster*. Science.

[50] Kazama H. (2015). Systems neuroscience in *Drosophila*: Conceptual and technical advantages. Neuroscience.

[51] Maximino C., Silva R.X., da Silva S.N., Rodrigues L.S., Barbosa H., de Carvalho T.S., Leão L.K., Lima M.G., Oliveira K.R., Herculano A.M. (2015). Non-mammalian models in behavioral neuroscience: Consequences for biological psychiatry. Front. Behav. Neurosci..

[52] Pandey U.B., Nichols C.D. (2011). Human disease models in *Drosophila melanogaster* and the role of the fly in therapeutic drug discovery. Pharm. Rev..

[53] Dong T., He J., Wang S., Wang L., Cheng Y., Zhong Y. (2016). Inability to activate Rac1-dependent forgetting contributes to behavioral inflexibility in mutants of multiple autism-risk genes. Proc. Natl. Acad. Sci. USA.

[54] Tian Y., Zhang Z.C., Han J. (2017). *Drosophila* Studies on Autism Spectrum Disorders. Neurosci. Bull..

[55] Koemans T.S., Oppitz C., Donders R.A.T., van Bokhoven H., Schenck A., Keleman K., Kramer J.M. (2017). *Drosophila* Courtship Conditioning As a Measure of Learning and Memory. J. Vis. Exp..

[56] Yoo H. (2015). Genetics of autism spectrum disorder: Current status and possible clinical applications. Exp. Neurobiol..

[57] Pramparo T., Pierce K., Lombardo M.V., Barnes C.C., Marinero S., Ahrens-Barbeau C., Murray S.S., Lopez L., Xu R., Courchesne E. (2015). Prediction of autism by translation and immune/inflammation coexpressed genes in toddlers from pediatric community practice. JAMA Psychiatry.

[58] Onore C., Yang H., Van de Water J., Ashwood P. (2017). Dynamic Akt/mTOR signaling in children with autism spectrum disorder. Front. Pediatr..

[59] Winden K.D., Ebrahimi-Fakhari D., Sahin M. (2018). Abnormal mTOR activation in autism. Annu. Rev. Neurosci..

[60] Sahin M., Sur M. (2015). Genes, circuits, and precision therapies for autism and related neurodevelopmental disorders. Science.

[61] Bou Khalil R. (2012). Is there any place for macrolides in mood disorders?. Med. Hypotheses.

[62] Kraushar M.L., Popovitchenko T., Volk N.L., Rasin M.R. (2016). The frontier of RNA metamorphosis and ribosome signature in neocortical development. Int. J. Dev. Neurosci..

